# Anatomical variations of the axilla

**DOI:** 10.1186/2193-1801-3-306

**Published:** 2014-06-24

**Authors:** Emerson Wander Silva Soares

**Affiliations:** Biological Science and Health Center, Western Paraná State University, (Universidade Estadual do Oeste do Paraná, UNIOESTE), Cascavel, Paraná, Brazil; Department of Surgical Oncology, Study and Treatment Cancer Center of Western Paraná, (União Oeste Paranaense de Estudos e Combate ao Câncer, UOPECCAN), Cascavel, Paraná, Brazil

**Keywords:** Axillary anatomy, Axillary lymph node dissection, Normative variation, Breast neoplasm

## Abstract

**Purpose:**

The present study aimed to measure the thickness of the subcutaneous adipose tissue (SAT) at the site of the surgical incision for axillary lymph node dissection (ALND) and to record potential anatomical variations in the medial cutaneous nerve of the arm (MCNA), the intercostobrachial nerve (ICBN), the lateral thoracic vein (LTV), the lateral thoracic artery (LTA) and the pectoral muscle pedicle (PMP), considering that some details of the anatomy of these structures within the axilla are still unclear.

**Methods:**

A prospective study was conducted in 100 consecutive patients with breast cancer who underwent ALND as part of surgical treatment. The anatomy of the dissected axilla was video recorded.

**Results:**

The SAT thickness ranged from 8 mm to 60 mm, with an average thickness of 25.9 mm. A positive correlation was observed between the SAT thickness and the body mass index (BMI) of the evaluated patients (r = 0.68; *p* < 0.0001). The MCNA was the anatomical structure that was least commonly observed in the axilla (22% of cases), while the PMP was the most constant element, identified in 100% of cases. All of the studied anatomical structures observed within the axilla showed variation in at least one of the aspects analyzed, i.e., the point of entry and exit, path, number and location of divisions or branches.

**Conclusion:**

The present study demonstrated wide variation in thickness of the SAT overlying the axilla and identified the existence of broad normative anatomical variation of the axilla.

**Electronic supplementary material:**

The online version of this article (doi:10.1186/2193-1801-3-306) contains supplementary material, which is available to authorized users.

## Introduction

Detailed knowledge of the anatomy of the axilla represents a basic foundation for surgeons who explore the axilla searching for sentinel lymph nodes or to perform conventional axillary lymph node dissection (ALND). Axillary lymph node dissection is usually performed for the staging or as a component of surgical treatment in patients with breast cancer (Pesce & Morrow 2013; Rao et al. 2013). According to estimates from the National Cancer Institute (NCI), 232,340 new cases of breast cancer were identified in the United States of America (USA) in 2013 alone (National Cancer Institute 2014).

The evolution of the axillary lymph node approach for the treatment of breast cancer, i.e., “en bloc” dissection of the axilla, initially advocated by Halsted (1907) in the early twentieth century for the sentinel lymph node biopsy (SLNB) (Veronesi et al. 2003), allowed the preservation of nerves and blood vessels closely related to the axillary lymph nodes. Even in cases with an indication for ALND, a “fragmented” dissection of the axilla may allow preservation of the neurovascular elements of this region (Ivanovic et al. 2008; Zhu et al. 2014).

The morbidities caused by ALND in the shoulder and arm, such as limited movement, pain, sensory changes and lymphedema (Warmuth et al. 1998; Keramopoulos et al. 1993; Ververs et al. 2001; Soares et al. 2014), are a result of unintentional damage to the lymphatic vessels, blood vessels and nerves interspersed with the dissected axillary lymph nodes. In a systematic review of 5,448 patients, Verbelen et al. (2014) found that in patients with breast cancer, even SLNB may result in chronic sequelae (two years after surgery), such as limitation of arm abduction (0-41.4%), pain (5.6-51.1%), paresthesia (5.1-51.1%) and lymphedema (0-27.3%). For this reason, the goal of refining the surgical technique for the dissection of axillary lymph nodes remains a current and recurring theme in the medical literature (Ung et al. 2006; Ponzone et al. 2009).

The surgeon’s knowledge of the presence and location of fascia, nerves and blood vessels as well as their relationship with other anatomical landmarks of the axilla has strategic importance for the identification and preservation of these structures in ALND and SLNB surgery. The nerves and vessels located along the anatomical boundaries of the axilla are routinely identified and were described and preserved long ago, including the long thoracic nerve (nerve of Bell) and the thoracodorsal pedicle (TDP) (Madden 1965). However, the literature contains little information on the location, path or possible branching of the following anatomical structures: the medial cutaneous nerve of the arm (MCNA), the intercostobrachial nerve (ICBN), the lateral thoracic vein (LTV), the lateral thoracic artery (LTA) and the pectoral muscle pedicle (PMP).

Therefore, the present study aimed to identify and describe the locations and interrelationships of the aforementioned vessels and nerves and to measure the thickness of the subcutaneous adipose tissue (SAT) at the site of the surgical incision performed to approach the axillary lymph nodes, which is usually coincident with or near the hairline.

## Methods

### Patient recruitment

A prospective study conducted at a single institution, the Study and Treatment Cancer Center of Western Paraná (União Oeste Paranaense de Estudos e Combate ao Câncer – UOPECCAN), located in the city of Cascavel, in the state of Paraná, which is a reference center for the treatment of cancer patients in southern Brazil. The present study was approved by the Committee for the Analysis of Research Projects of UOPECCAN and the Research Ethics Committee of Western Paraná State University (Universidade Estadual do Oeste do Paraná - UNIOESTE). All participants signed an informed consent form. In total, 100 consecutive patients, from July 2012 to August 2013, were evaluated. The patients had invasive breast cancer and underwent ALND at Berg levels I and II (Berg 1955) as part of their surgical treatment. The sample size was determined by the previously established number of cases necessary to evaluate normative variations (Bridges & Holler 2007).

### Data collection

The arrangement and relationship between the anatomical structures preserved at the end of ALND was determined by video recording the dissected axilla for approximately one minute, immediately before the surgical wound was closed. A Sony® HDR-PJ260V handycam was used for this purpose. To record the images, the participants in the study were positioned with their arm on the side of the surgery abducted at 90°, resting on a support. The flaps that cover the axilla were moved away with surgical instruments. The thickness of the SAT in the flap that covers the axilla at the site of the surgical incision was assessed and recorded in millimeters using a ruler when this surgical plan was opened. The deep limit of the SAT was delimited by identifying the axillary fascia.

### Nomenclature of the identified anatomical structures

The anatomical structures discussed in the present study are: MCNA, ICBN, LTV, LTA and PMP. They were chosen due to its potential of variability, identified and referred to as described in Gray’s anatomy atlas (Standring 2008) and in previously published articles that have addressed this subject (Loukas et al. 2006; Khan et al. 2012; Nadkarni & Raina 2006; Macéa & Fregnani 2006). To obtain a better understanding, ICBN was divided into three distinct anatomical structures: the first ICBN, identified from the second intercostal space, at the top of the axilla, originating in the second intercostal nerve (T2); the second ICBN, identified from the third intercostal space and originating in the third intercostal nerve (T3); and the third ICBN, identified from the fourth intercostal space in the caudal region of the axilla and originating from the fourth intercostal nerve (T4).

### Statistical analysis

The data are presented as the range, means and standard deviation (±) for numerical variables. The correlation between SAT thickness and the body mass index (BMI) was determined via Pearson’s linear correlation. P values < 0.05 were considered significant. The statistical treatment of the data was performed using the BioEstat® software statistical package, version 5.3; available at: http://www.mamiraua.org.br/.

## Results

The anatomical variations of 100 axillae dissected for lymph node resection as part of the surgical treatment of patients with breast cancer were recorded. Among the recruited patients, 99 were female and one was male. The right axilla was dissected in 52 cases, and the left axilla was dissected in 48 cases. The BMI was determined for all 100 patients and ranged from 17.4 to 43.7, with a mean value of 27.6 ± 5.18.

The anatomical variations evaluated in the present study are described below.

1. Subcutaneous adipose tissue (SAT) thickness

**Figure 1:**
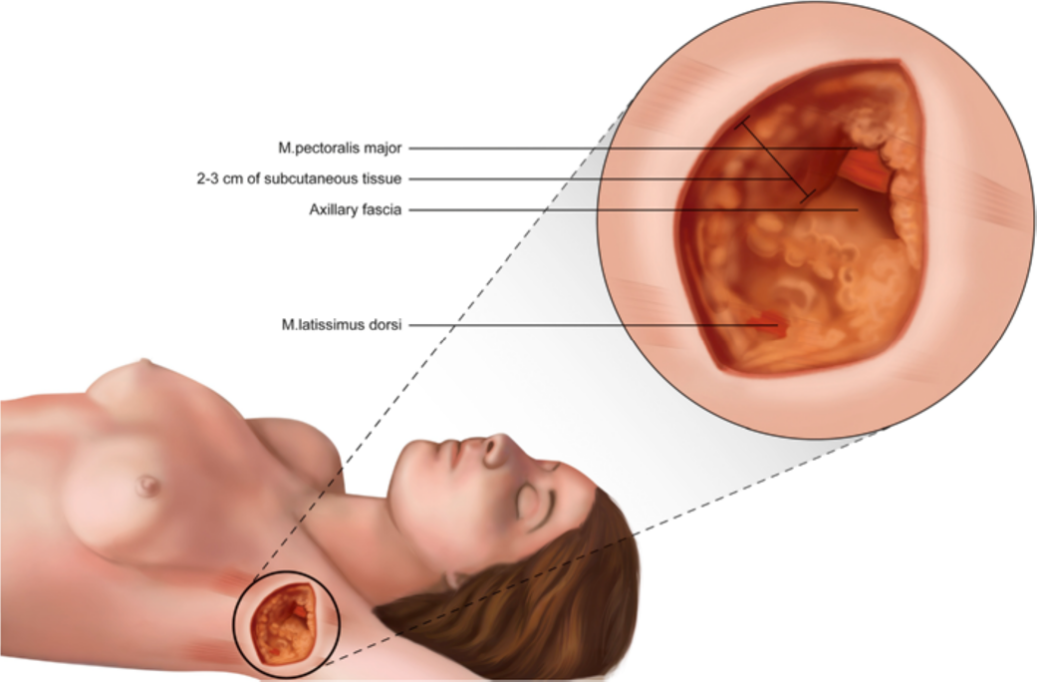
**Illustration of the subcutaneous adipose tissue (SAT) thickness at the site where the surgical incision (hairline) is commonly made to perform axillary lymph node dissection.**

The SAT thickness could not be determined in seven cases from the total of 100 cases evaluated because the axillary fascia was not identified. In the 93 cases in which this measurement was possible, the SAT thickness in the surgical incision ranged from 8 mm to 60 mm, with a mean of 25.9 mm (SD ± 8.45). There was a strong positive correlation between the BMI and the thickness of the flap according to Pearson’s linear correlation parametric statistical test (r = 0.68, *p* < 0.0001). In other words, as the BMI increases, so does SAT thickness (Figure 1; Additional file 1A).

2) Medial cutaneous nerve of the arm (MCNA); Cutaneus brachii medialis nerve

This nerve was identified in 22% (n = 22) of the cases evaluated. The MCNA most commonly penetrates the axilla through the apex (n = 13; 59.1%), along with the axillary vein, and crosses the axilla just below and close to this vein (lower and parallel). Another presentation of the MCNA is an initial path posterior to the axillary vein, followed by its appearance behind the axillary vein and visualization below the axillary vein until it exits the axilla (n = 9; 40.9%) toward the arm (Additional file 1B). In most of the cases in which this nerve was identified, the MCNA did not communicate with the first ICBN (63.6%; n = 14). However, in 36.4% of the cases (n = 8), it communicated with the first ICBN. In these situations, the MCNA deviated from the axillary vein during its path through the axilla. In three cases (13.6%), before reaching the arm, the MCNA split into two branches during its path through the axilla.

**Figure 2:**
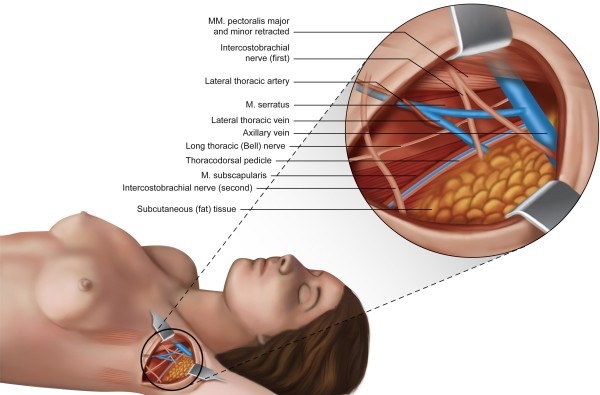
**Illustration showing an example of the distribution of the anatomical structures identified within the axilla after axillary lymph node dissection, in which the first and second ICBN are present.** The first ICBN splits into two branches in the central region of the axilla.

3) First intercostobrachial nerve (T2) – ICBN; Intercostobrachialis nerveThis structure was identified and was preserved in at least one branch until reaching the arm in 99 of the 100 cases evaluated. In one case, the first ICBN was not identified, and only the second ICBN was found. The ICBN crossed the axilla with no divisions in 26.2% (n = 26) of the 99 cases in which it was identified; in 33.3% (n = 33) of the cases, the ICBN split into branches in the first 2 centimeters of the path in the axilla (n = 23) or entered the axilla already split into two branches (n = 9) or up to three branches (n = 1). In 40.4% of the cases (n = 40) divisions of the ICBN were observed in the central region of the axilla (Figure 2) and in 20.2% (n = 20) of the cases, the ICBN split in the last two centimeters before leaving the axilla toward the arm. The sum of the cases described is not 99 because in 19 cases, the first ICBN split more than once on the path through the axilla. In 14 cases (14.1%), branches of the first ICBN were sectioned.

4) Second intercostobrachial nerve (T3) ICBN; Intercostobrachialis nerve

The second ICBN was identified in 61% (n = 61) of the cases. This structure communicated with the first ICBN in only three cases (4.9%), and its path through the axilla toward the arm was parallel to and lower than that of the first ICBN. The second ICBN showed no branches in 45 cases (73.7%). Branching of the second ICBN was observed in 19 cases (in three cases, there were branches in more than one segment of the path). Among these cases, 14 either presented a division that occurred earlier, within the first two centimeters of the path in the axilla, or the second ICBN entered the axilla already divided into three distinct branches (n = 1) (Additional file 1C). In two cases, a split was observed in the central region of the axilla, and in two cases, such a split occurred in the final two centimeters of the path in the axilla. The second ICBN was sectioned, in its trunk or branches, in 13 of the 61 cases in which it was identified (21.3%).

5) Third intercostobrachial nerve (T4) ICBN; Intercostobrachialis nerve

The third ICBN was identified in three (3%) of the 100 cases evaluated. This structure did not exhibit branching and did not communicate with other nerves. Its path in the axilla, toward the arm, was parallel to and lower than the path of the second ICBN. The third ICBN was preserved in the three cases in which it was identified (Additional file 1C).

**Figure 3:**
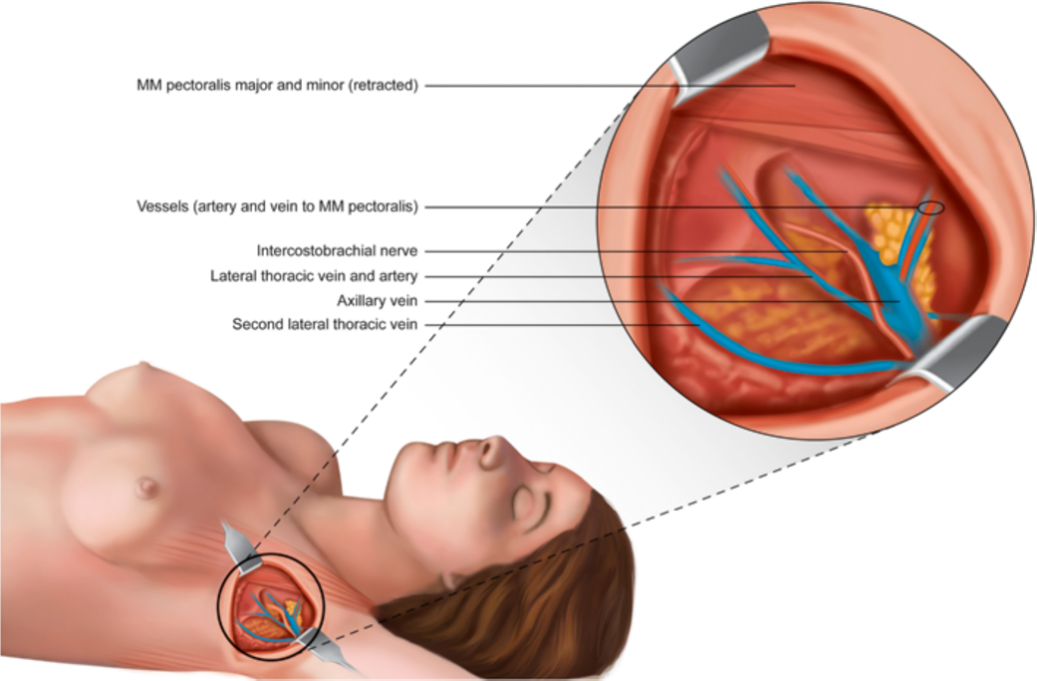
**Illustration of an anatomical variation of the axilla in which a pectoral muscle pedicle formed by two veins and one artery and the presence of two independent lateral thoracic veins are observed.**

6) Lateral thoracic vein (LTV); Thoracica lateralis veinThe LTV was identified in 98 of the 100 cases evaluated. In one case, the LTV was duplicated across its entire path through the axilla, showing independent origins and terminations and with no communication between the duplicate structures (Figure 3). In 61.2% of the cases in which it was identified (n = 60), the LTV received no tributary branches within its path, which was vertical, upward and parallel to the chest wall, from its point of entry near the base of the axilla, to its direct drainage into the axillary vein. In 38.8% (n = 38) of the examined cases, the LTV received one or more branches from the chest wall (17.4%; n = 17), the arm (7.1%; n = 7) or both regions (14.3%; n = 14). The junction between the LTV and the axillary vein always occurred prior to and separate from the TDP. In 39.4% (n = 39) of the cases, the LTV drained into the axillary vein anteromedial to the TDP. In 36.4% (n = 36) of the cases, the LTV drained into the axillary vein in the same plane as the TDP. In 24.2% (n = 24) of the cases, the LTV drained into the axillary vein anterolateral to the TDP, and the path was vertical and showed a medial-to-lateral direction within the axilla. The total number of drainages observed was 99 because in one case, two axillary veins that drained independently were observed in the same plane of the TDP and anterolateral to the TDP. In 56.1% (n = 55) of the cases evaluated, the LTV was joined by the LTA in its path through the axilla, whereas in 43.9% (n = 43) of the cases, the LTV was not joined by this artery.

7) Lateral thoracic artery (LTA); Thoracica lateralis arteryThe LTA was identified in 55 of the 100 cases evaluated. Its path was always close to the LTV. In some cases, the LTA was wrapped around the LTV and partly covered by it (Figure 2). No branches were observed in its path through the axilla in the 55 cases in which it was identified.
8) Pectoral muscle pedicle (PMP)

The PMP was identified in 100% of the cases evaluated (n = 100) and was preserved in 99% of the cases (n = 99). The PMP forms a loop from the axillary vein and the axillary artery to the lateral edge of the pectoralis minor muscle. Regarding the composition of the PMP, the presence of one artery and one vein was observed in 91 cases (91%). In nine patients (9%), two veins and one artery were identified (Figure 3). None of the cases showed the medial pectoral nerve within the composition of the PMP (Additional file 1D).

## Discussion

The routine surgical approach to the axilla for lymph node dissection in patients with breast cancer drew surgeon attention for two common facts: (1) vessels and nerves not identified in the locations described in anatomy atlases in some cases, and in others, (2) vessels and nerves identified in unexpected locations. Even considering that such modifications of the anatomy or of the “pyramidal” form of the axilla can be explained by changes in the position of the arm (Ger & Kim 1988), the hypothesis that there is normative variation of the anatomy of the axilla, which is still poorly studied in the medical literature, seemed justifiable.

Searching for uncommon anatomical changes, such as the presence of abnormal muscles or tendons (Natsis et al. 2010; Besana-Ciani & Greenall 2005) or duplication of the axillary vein (Kutiyanawala et al. 1998) was not part of the objectives of the present study.

Regarding the thickness of the SAT at the site where the axilla was opened, wide variation (8 mm to 60 mm) and correlation with the BMI were observed among the evaluated patients. This correlation is not observed for the SAT that covers the breast, as reported by Larson et al. (2011). Additionally, the average thickness of the SAT that covers the breast, as described by these authors (Larson et al. 2011), is 1 cm, which is much thinner compared with the average thickness of the SAT in the axillary region observed in the present study (25.9 mm). Therefore, knowing this difference is important because maintaining the same flap thickness in the breast and axillary regions may result in unnecessary removal of the SAT overlying the axillary fascia. Furthermore, it can result in accidental damage to the superficial lymphatic vessels located in the SAT (Suami et al. 2007a) as well as in damage to the anastomoses present in this region between the superficial and deeper lymphatic vessels that drain lymph from the upper member (Suami et al. 2007b; Pavlista & Eliska 2012), which theoretically may contribute to an increased risk of lymphedema of the arm. Although the optimum thickness of the flap after skin-sparing mastectomy for treatment of breast cancer has been the subject of numerous studies (Torresan et al. 2005; Carlson 2011) and controversies (Bleicher et al. 2003) because of the risk of local recurrence, determining the thickness of the flap at the surgical incision site for approaching the axillary lymph nodes is a topic that has not been addressed in the literature. The axillary fascia, which is an anatomical landmark that separates the SAT from the axilla itself at its base, is relatively thick and has been identified with “the naked eye” in the vast majority (93%) of cases evaluated.

The MCNA, which is responsible for the sensitivity of the medial distal third of the arm (brachial plexus branch – C8, T1), was not identified within the axilla in most of the cases (78%) evaluated in the present study. When this nerve was identified, it was most common that it crosses the axilla parallel and anterior to and running alongside the axillary vein throughout its course (59.1%). Communication between the MCNA and ICBN was not as frequent (36.4%) as observed by Race and Saldana (90%) (Race & Saldana 1991), nor was its division into branches along the path through the axilla before reaching the arm (13.6%). The importance of this knowledge lies in the fact that the MCNA is not always protected and posterior to the axillary vein. Therefore, it is subject to injury during the dissection of axillary lymph nodes, particularly when performing dissections of Berg level III, or during dissections of levels I and II in cases where the MCNA deviates from the axillary vein to communicate with the first ICBN.

The results obtained in the present study show that up to three intercostobrachial nerves may pass through the axilla simultaneously at different levels: the first ICBN in the cranial position, the second in the central region and the third in the caudal region of the axilla. The communication between the intercostobrachial nerves described by Loukas et al. (2006) was observed in only three cases between the branches of the first and second ICBN. Conversely, the division of the first and second ICBN into one or more branches within the axilla was a common finding in the present study, and in some cases, the first and second ICBN emerged in the axilla already having split into two or three branches, which increases the difficulty of their preservation. The ICBN is responsible for the sensitivity of the internal and proximal regions of the arm. Studies present conflicting results regarding the benefit of preserving the ICBN in ALND. Salmon et al. (1998) observed no benefits regarding the sensitivity of the arm when preserving the ICBN. Abdullah et al. (1998) reported that sectioning of the ICBN does not always result in loss of sensation in the arm. Freeman et al. (2003) demonstrated that a possible sensory loss in the arm after sectioning the ICBN was usually transitory. However, several studies, including a meta-analysis, have demonstrated a significant sensory benefit when the ICBN is preserved (Ivanović et al. 2007; Warrier et al. 2014). Based on the findings of the present study, should be considered the hypothesis that the extent and severity of sensory loss in the arm are directly proportional to the size of the lesion caused in the ICBN, which remains to be confirmed in future studies. As demonstrated in the present study, the preservation or sectioning of a nerve branch that crosses the axilla toward the arm does not mean that the ICBN is *fully* preserved or sectioned.

The LTV was was a constant anatomical structure in the axilla. The same cannot be said for the LTA, which was absent in 45% of the cases evaluated. Most commonly, drainage of the LTV into the axillary vein anteromedial to the TDP was observed, confirming the findings of Khan et al. (2012). However, in all of the cases studied, there was no communication between the LTV and the subscapular vein before the axillary vein was reached. In 24.2% of the cases evaluated in the present study, the LTV shifted away from the chest wall and crossed the central region of the axilla to reach the axillary vein in its most distal portion, near the arm. Because of this finding and considering the variations in the position of the ICBN previously described, the anatomical classification of the axilla based on the intersection between the ICBN and LTV that was proposed by Clough et al. (2010) for locating the sentinel lymph node could not be applied in a considerable percentage of patients in the present study.

The pectoral muscle pedicle, referred to as the “medial pectoral pedicle” by Nadkarni & Raina (2006), was also a constant anatomical structure in the present study, being observed in 100% of the evaluated cases and composed of an artery and a vein in 91 cases (91%). In nine cases, two veins and one artery were identified. The presence of a nerve structure was not observed in any of the cases studied, unlike what was described by Nadkarni & Raina (2006). One possible explanation for not identifying the medial pectoral nerve in the present study is its most medial location in the axilla (Berg level III) and the path between the pectoral muscles, as described by Moosman (1980).

In conclusion, the present study identified and described anatomical details of the axilla that have not previously been published in the medical literature and demonstrated the existence of wide normative variation in the anatomy of the axilla, with no pretense of exhausting the subject.

## Electronic supplementary material

Additional file 1A, 1B, 1C and 1D: **Anatomial variations of the axilla.** (MPEG 206 MB)

## Abbreviations

SLNB: Sentinel lymph node biopsy
ALND: Axillary lymph node dissection
MCNA: Medial cutaneous nerve of arm
ICBN: Intercostobrachial nerve
LTV: Lateral thoracic vein
LTA: Lateral thoracic artery
PMP: Pectoral muscles pedicle
TDP: Thoracodorsal pedicle
SAT: Subcutaneous adipose tissue
BMI: Body mass index.

## References

[CR1] Abdullah TI, Iddon J, Barr L, Baildam AD, Bundred NJ (1998). Prospective randomized controlled trial of preservation of the intercostobrachial nerve during axillary node clearance for breast cancer. Br J Surg.

[CR2] Berg JW (1955). The significance of axillary node levels in the study of breast carcinoma. Cancer.

[CR3] Besana-Ciani I, Greenall MJ (2005). Langer's axillary arch: anatomy, embryological features and surgical implications. Surgeon.

[CR4] Bleicher RJ, Hansen NM, Giuliano AE (2003). Skin-sparing mastectomy. Specialty bias and worldwide lack of consensus. Cancer.

[CR5] Bridges AJ, Holler KA (2007). How many is enough? Determining optimal samples size for normative studies in pediatric neuropsychology. Child Neuropsychol.

[CR6] Carlson GW (2011). Technical advances in skin sparing mastectomy. Int J Surg Oncol.

[CR7] Clough KB, Nasr R, Nos C, Vieira M, Inguenault C, Poulet B (2010). New anatomical classification of the axilla with implications for sentinel node biopsy. Br J Surg.

[CR8] Freeman SR, Washington SJ, Pritchard T, Barr L, Baildam AD, Bundred NJ (2003). Long term results of a randomised prospective study of preservation of the intercostobrachial nerve. Eur J Surg Oncol.

[CR9] Ger R, Kim D (1988). An aid to axillary dissection. Surg Gynecol Obstet.

[CR10] Halsted WS (1907). The results of radical operations for the cure of carcinoma of the breast. Ann Surg.

[CR11] Ivanović N, Granić M, Randelović T, Bilanović D, Dukanović B, Ristić N, Babić D (2007). Functional effects of preserving the intercostobrachial nerve and the lateral thoracic vein during axillary dissection in breast cancer conservative surgery. Vojnosanit Pregl.

[CR12] Ivanovic N, Granic M, Randjelovic T, Todorovic S (2008). Fragmentation of axillary fibrofatty tissue during dissection facilitates preservation of the intercostobrachial nerve and the lateral thoracic vein. Breast.

[CR13] Keramopoulos A, Tsionou C, Minaretzis D, Michalas S, Aravantinos D (1993). Arm morbidity following treatment of breast cancer with total axillary dissection: a multivariated approach. Oncology.

[CR14] Khan A, Chakravorty A, Gui GP (2012). In vivo study of the surgical anatomy of the axilla. Br J Surg.

[CR15] Kutiyanawala MA, Stotter A, Windle R (1998). Anatomical variants during axillary dissection. Br J Surg.

[CR16] Larson DL, Basir Z, Bruce T (2011). Is oncologic safety compatible with a predictably viable mastectomy skin flap?. Plast Reconstr Surg.

[CR17] Loukas M, Hullett J, Louis RG, Holdman S, Holdman D (2006). The gross anatomy of the extrathoracic course of the intercostobrachial nerve. Clin Anat.

[CR18] Macéa JR, Fregnani JHTG (2006). Anatomy of the thoracic wall, axilla and breast. Int J Morphol.

[CR19] Madden JL (1965). Modified radical mastectomy. Surg Gynecol Obstet.

[CR20] Moosman DA (1980). Anatomy of the pectoral nerves and their preservation in modified mastectomy. Am J Surg.

[CR21] Nadkarni MS, Raina S (2006). Badwe RA (2006) Medial pectoral pedicle: a critical landmark in axillary dissection. ANZ J Surg.

[CR22] (2014). Breast Cancer.

[CR23] Natsis K, Vlasis K, Totlis T, Paraskevas G, Noussios G, Skandalakis P, Koebke J (2010). Abnormal muscles that may affect axillary lymphadenectomy: surgical anatomy. Breast Cancer Res Treat.

[CR24] Pavlista D, Eliska O (2012). Analysis of direct oil contrast lymphography of upper limb lymphatics traversing the axilla – A lesson from the past – Contribution to the concept of axillary reverse mapping. Eur J Surg Oncol.

[CR25] Pesce C, Morrow M (2013). The need for lymph node dissection in nonmetastatic breast cancer. Annu Rev Med.

[CR26] Ponzone R, Cassina E, Tomasi Cont N, Biglia N, Sismondi P (2009). Decreasing arm morbidity by refining axillary surgery in breast cancer. Eur J Surg Oncol.

[CR27] Race CM, Saldana MJ (1991). Anatomic course of the medial cutaneous nerves of the arm. J Hand Surg [Am].

[CR28] Rao R, Euhus D, Mayo HG, Balch C (2013). Axillary node interventions in breast cancer: a systematic review. JAMA.

[CR29] Salmon RJ, Ansquer Y, Asselain B (1998). Preservation versus section of intercostal-brachial nerve (IBN) in axillary dissection for breast cancer – a prospective randomized trial. Eur J Surg Oncol.

[CR30] Soares EW, Nagai HM, Bredt LC, da Cunha AD, Andrade RJ, Soares GV (2014). Morbidity after conventional dissection of axillary lymph nodes in breast cancer patients. World J Surg Oncol.

[CR31] Standring S (2008). Gray’s Anatomy: the Anatomical Basis of Clinical Practice (40th edn).

[CR32] Suami H, Taylor GI, Pan WR (2007). The lymphatic territories of the upper limb: anatomical study and clinical implications. Plast Reconstr Surg.

[CR33] Suami H, Pan WR, Taylor GI (2007). Changes in the lymph structure of the upper limb after axillary dissection: radiographic and anatomical study in a human cadaver. Plast Reconstr Surg.

[CR34] Torresan RZ, dos Santos CC, Okamura H, Alvarenga M (2005). Evaluation of residual glandular tissue after skin-sparing mastectomies. Ann Surg Oncol.

[CR35] Ung O, Tan M, Chua B (2006). Barraclough B (2006) Complete axillary dissection: a technique that still has relevance in contemporary management of breast cancer. ANZ J Surg.

[CR36] Verbelen H, Gebruers N, Eeckhout FM, Verlinden K, Tjalma W (2014). Shoulder and arm morbidity in sentinel node-negative breast cancer patients: a systematic review. Breast Cancer Res Treat.

[CR37] Veronesi U, Paganelli G, Viale G, Luini A, Zurrida S, Galimberti V, Intra M, Veronesi P, Robertson C, Maisonneuve P, Renne G, De Cicco C, De Lucia F, Gennari R (2003). A randomized comparison of sentinel-node biopsy with routine axillary dissection in breast cancer. N Engl J Med.

[CR38] Ververs JM, Roumen RM, Vingerhoets AJ, Vreugdenhil G, Coebergh JW, Crommelin MA, Luiten EJ, Repelaer van Driel OJ, Schijven M, Wissing JC, Voogd AC (2001). Risk, severity and predictors of physical and psychological morbidity after axillary lymph node dissection for breast cancer. Eur J Cancer.

[CR39] Warmuth MA, Bowen G, Prosnitz LR, Chu L, Broadwater G, Peterson B, Leight G, Winer EP (1998). Complications of axillary lymph node dissection for carcinoma of the breast: a report based on a patient survey. Cancer.

[CR40] Warrier S, Hwang S, Koh CE, Shepherd H, Mak C, Carmalt H, Solomon M: **Preservation or division of the intercostobrachial nerve in axillary dissection for breast cancer: Meta-analysis of Randomised Controlled Trials.** *Breast* 2014., (14)**:** doi: 10.1016/j.breast.2014.01.014. [Epub ahead of print] 10.1016/j.breast.2014.01.014 24582033

[CR41] Zhu JJ, Liu XF, Zhang PL, Yang JZ, Wang J, Qin Y, Zhang GL, Ren DQ, Cui CL, Guo XG (2014). Anatomical information for intercostobrachial nerve preservation in axillary lymph node dissection for breast cancer. Genet Mol Res.

